# Peptides as Potentially Anticarcinogenic Agent from Functional Canned Meat Product with Willow Extract

**DOI:** 10.3390/molecules27206936

**Published:** 2022-10-16

**Authors:** Karolina M. Wójciak, Paulina Kęska, Monika Prendecka-Wróbel, Karolina Ferysiuk

**Affiliations:** 1 Department of Animal Food Technology, Faculty of Food Science and Biotechnology, University of Life Sciences in Lublin, Skromna 8, 20-704 Lublin, Poland; 2 Chair and Department of Human Physiology, Medical University of Lublin, Radziwiłłowska 11, 20-080 Lublin, Poland

**Keywords:** nitrite replacement, health, *E. angustifolium*, cell lines

## Abstract

The aim of the study was to demonstrate canned pork as a functional meat product due to the presence of potentially anti-cancer factors, e.g., (a) bioactive peptides with potential activity against cancer cells; (b) lowering the content of sodium nitrite and with willow herb extract. In silico (for assessing the anticancer potential of peptides) and in vitro (antiproliferation activity on L-929 and CT-26 cell lines) analysis were performed, and the obtained results confirmed the bioactive potential against cancer of the prepared meat product. After 24 h of incubation with peptides obtained from meat product containing lyophilized herb extract at a concentration of 150 mg/kg, the viability of both tested cell lines was slightly decreased to about 80% and after 72 h to about 40%. On the other hand, after 72 h of incubation with the peptides obtained from the variant containing 1000 mg/kg of freeze-dried willow herb extract, the viability of intestinal cancer cells was decreased to about 40%, while, by comparison, the viability of normal cells was decreased to only about 70%.

## 1. Introduction

Meat is rich in essential nutrients, including protein, fat, fatty acids, vitamins, and minerals, and is, hence, considered as an important nutritional component of the human diet. However, it may also contain harmful chemical compounds, such as heterocyclic amines, polycyclic aromatic hydrocarbons, nitrates, and N-nitroso compounds, which have been shown to increase the risk of cancer-related morbidity. This poses a significant challenge to producers and consumers, as malignant neoplasms, which are one of the most common noncommunicable diseases, have been increasing worldwide. According to the report of the International Agency for Research on Cancer (IARC) published in 2015 [1], more than 800 studies from the last 20 years have pointed out the relationship between meat consumption and the incidence of cancer. As its regular consumption has been linked with cancer development, and in line with the IARC opinion, red meat has been identified as potentially carcinogenic and classified under Group 2A. Processed meat has been found to be a much more potent carcinogen and is, therefore, classified as Group 1, which includes factors with compelling evidence of human carcinogenicity. As a result, the World Cancer Research Fund (WCRF) recommended that the consumption of red meat should be limited to <500 g per week and processed meat should be avoided as much as possible [2]. The relationship between the consumption of red meat and processed meat and cancer development has been widely described in the literature. Red meat consumption has been shown to increase the risk of bladder cancer, breast cancer, colorectal cancer, endometrial cancer, esophageal cancer, stomach cancer, lung cancer, nasopharyngeal cancer, non-Hodgkin’s lymphoma, and overall cancer mortality. Similarly, the consumption of processed meat has been associated with a higher risk of bladder cancer, breast cancer, colorectal cancer, esophageal cancer, gastric cancer, nasopharyngeal cancer, non-Hodgkin’s lymphoma, mouth and oropharyngeal cancer, prostate cancer, and overall cancer mortality [3].

Colorectal cancer, in particular, is a major concern, accounting for 10% of all cancer diagnoses (ranking third among the most commonly diagnosed cancers) and 9.4% of all cancer deaths in 2020 (ranking second for cancer mortality). Singh and Fraser [4] indicated that individuals who consumed pork or beef once a week had a 90% higher risk of colon cancer, in comparison to those who were on a meat-free diet. English et al. [5] proved that participants who consumed red meat almost every day (>6.5 times a week) had a 40% higher risk of colorectal cancer and 130% higher risk of rectal cancer, compared to those who consumed less meat (<3 times per week). Furthermore, Norat et al. [6] highlighted that consumption of 100 g of red meat everyday increased the risk of colorectal cancer by 21%. Currently, a wide range of treatment options are available for colorectal cancer. However, these often lead to trauma and side effects, cause damage to healthy cells, and are expensive. Thus, there is a search for new treatments for colon cancer. Recent studies show that functional foods loaded with natural compounds can prevent the risk of developing colon cancer. Therefore, in this study, a meat product with functional properties was developed, in which (a) the content of sodium nitrite was reduced to 50 mg/kg to reduce the risk of formation of carcinogenic compounds and (b) phytochemicals in the form of extracts rich in polyphenols obtained from willow herb, which is known for its strong bioactive properties, including as an antioxidant, anti-inflammatory, anti-cancer. The strategy to halve sodium nitrate is an action to reduce its potentially carcinogenic nature. Eliminating sodium nitrate or reducing the recommended amount to half (from 100 mg/kg to 50 mg/kg) is associated with losses in the quality and safety of canned pork [7], but it turned out to be possible by fortifying low-nitrite canned meat with a lyophilized extract from willow herbs [8]. In addition, our previous studies [9] have shown that the addition of E. angustifolium extracts has an impact on the peptide profile and their various bioactivities. In particular, the addition of herb at the level of 150 mg/kg enhanced the antioxidant effect of peptides from canned pork. Indeed, meat and meat products are a good source of biologically active peptides with various health-promoting effects, including anticarcinogenic properties, and thus can contribute to improving the effectiveness of anticancer therapy. The activity of such peptides may be related to their ability to inhibit angiogenesis and initiate necrosis or apoptosis, distort peptides needed for tumor cell proliferation, delay the activity of enzymes involved in tumor development, or increase immunity against tumor cells [10]. Protein hydrolysates or peptides derived from milk, eggs, fish, crabs, shrimps, sea cucumber, oysters, clams, chlorella (algae), spirulina, rice, soybeans, corn, beans, chickpeas, and rapeseed have been proven to have anticarcinogenic potential [11,12,13,14,15,16,17,18,19,20,21,22,23]. Furthermore, previous studies have shown that sausages and whole-muscle products (loins, sirloins, neck) contain biologically active peptides with primarily antioxidant and/or antihypertensive (in vitro) and anticarcinogenic (in silico) properties. Thus far, no study has been performed that investigates the anticarcinogenic potential of canned sterilized pork.

It should be noted that proteolytic digestion of food proteins may involve the formation of anticarcinogenic peptides, which can contribute to strengthening the body’s natural defense barrier. Moreover, such food proteins have protective effects against various types of pathogens, as indicated by scientific reports on the anticancer and antimicrobial effects of food peptides [21,24,25]. On the other hand, enzymatic hydrolysis can lead to a loss of the bioactive effect of the peptides due to too extensive degradation of the peptide chains. Therefore, in this study, the anticancer potential of peptides was determined in two stages: (a) the peptides obtained from canned pork were identified by spectrophotometric methods, and their anticarcinogenic effect was analyzed in silico, and (b) in vitro analysis of the antiproliferative properties of protein and peptide hydrolysates with enzymes of the gastrointestinal tract (pepsin and pancreatin) using L-929 and CT-26 cell lines.

The aim of this study was to demonstrate the functional nature of canned meat having reduced the content of sodium nitrite and to evaluate the potentially anticarcinogenic properties of canned meat peptides containing an aqueous, freeze-dried extract of willow herb at an amount of 150 mg/kg (E1) or 1000 mg/kg (E2). The potential of peptides as anticancer agent in the products were evaluated after 6 months of refrigerated (4 °C) storage based on in silico and in vitro analysis.

## 2. Materials and Methods

### 2.1. Preparation of Epilobium angustifolium L. extract and Lyophilization

Crushed, dried leaves of willow herb were used (herbal enterprise “Polskie Zioła”) to prepare water extracts. First, the plant material was immersed in redistilled water (90 °C ± 5 °C) in a ratio of 1:10 (g of sample: mL of solvent). The infusions were placed in an ultrasonic bath for extraction using Sonic 6D equipment (Polsonic Palczynki Sp. J., Poland). The ultrasound frequency was set as 40 kHz and sound intensity as 320 W/cm^2^, temperature as 30 °C, and extraction time as 10 min. After 30 min of extraction of the sample with water, the extracts were filtered through a funnel with filter paper. The filter cake was re-extracted under the same conditions, and the collected filtrates were frozen. Then, the frozen extracts were lyophilized using a Labconco FreeZone freeze dryer (Kansas City, MO, USA) for 72 h at −80 °C and 0.04 mbar. The lyophilized material was stored in tightly closed containers.

### 2.2. Preparation of Canned Pork Meat

Canned pork used in the study was prepared from pork dewlap and pork shoulder in a ratio of 2:8. The muscle was excised in a slaughterhouse (Zakład Mięsny Wasąg sp. J., organic certificate no. PL-EKO-093027/18) after a 48 h slaughter. The raw meat was precrushed with a knife and finely minced using a meat grinder (Ø 5 mm). The minced meat was divided into three individual variants, to which sodium chloride (2%), water (5%), or sodium nitrite (50 or 100 mg/kg) was added. The test variants were as follows: variant added with sodium nitrite at an amount of 50 mg/kg of stuffing and variant added with lyophilized willow herb extract at an amount of 150 mg/kg (E1) or 1000 mg/kg (E2). The control sample (C) contained twice the amount of sodium nitrate (100 mg/kg) and no plant extract. The remaining ingredients were added in equal amounts. The meat stuffing, thus, prepared was mixed using a universal machine-type KU2-3E device (Mesko-AGD, Poland; 5 min/variant) and transferred to metal cans. The batch weight was approximately 250 g. The metal cans were closed and placed in a vertical steam sterilizer (TYP-AS2) and subjected to sterilization (121 °C). The sterilized cans were cooled with cold water and stored in a refrigerator (4 °C) for 180 days.

### 2.3. Extraction and Spectrometric Identification of Peptides

Peptides were isolated from canned meat samples using the method proposed by Escudero et al. [26]. The resulting supernatant was dried in a vacuum evaporator (Rotavapor R-215, Büchi Labortechnik AG, Flawil, Switzerland). The dried extract was dissolved in a 0.01 N HCl solution, and the mixture was filtered through a 0.45 μM nylon membrane filter (Millipore, Bedford, MA, USA) and stored at −60 °C until further use. Peptide analysis was performed by liquid chromatography coupled with tandem electrospray mass spectrometry, as described in our previous study.

### 2.4. Sample Extraction and Gastrointestinal Digestion

After 180 days of storage, the meat samples were treated with proteases (pepsin and pancreatin), which are equivalent to human digestive enzymes. Briefly, proteins were obtained from samples by homogenizing them (10 mg) with a phosphate-buffered solution (15.6 mM Na_2_HPO_4_, 3.5 mM KH_2_PO_4_, pH 7.5) in a homogenizer (IKA T25, Staufen, Germany) at 8000 rpm for 5 min in a bath. The obtained homogenate was centrifuged at 5000× *g* for 20 min at 4 °C. The protein content of the extracts was determined by the Bradford method, using bovine serum albumin as reference. The resulting supernatant was hydrolyzed, as described by Escudero et al. [26]. Before hydrolysis, the pH of the extracts was adjusted to 2.0–2.5 with 1 M HCl. Then, pepsin was added (enzyme:protein solution 1:100) to the extracts for 2 h at 37 °C under constant stirring. The solution was neutralized to pH 7.0 (with 1 M NaOH), and then pancreatin was added (enzyme:protein solution 1:50) under the above-mentioned conditions. After hydrolysis, the samples were heated at 100 °C for 10 min. Peptides were isolated from the hydrolysates by ultrafiltration (7 kDa molecular weight cut off; Spectra/Por^®^; 1:4 dilution, phosphate-buffered saline (pH 7.4)). The collected samples were filtered through a 0.45 μM nylon membrane filter (Millipore, Bedford, MA, USA) and stored at −60 °C. The frozen peptides were lyophilized using a LabconcoFreeZone freeze dryer for 72 h at −80 °C and 0.04 mbar. The lyophilized material was stored in tightly closed containers. This material was assessed for antiproliferative activity using cell lines.

### 2.5. Anticancer Activity Prediction—In Silico Study

The peptides isolated from meat products after 180 days of refrigerated storage were subjected to in silico analysis. Peptide sequences were identified by spectrometric methods and then screened for fragments with anticancer activity using the iACP tool, which is a sequence-based tool available online for identifying anticancer peptides (http://lin-group.cn/server/iACP (accessed on 1 December 2021)). The amino acid composition of the peptides was determined using ProtParam (https://web.expasy.org/protparam/ (accessed on 1 December 2021)). Selected physicochemical properties, including hydrophobicity and net charge, as well as toxic properties of peptides were determined using ToxinPred (http://crdd.osdd.net/raghava/toxinpred/ (accessed on 1 December 2021)), and allergenicity was tested using AllerTOP v. 2.0 (https://www.ddg-pharmfac.net/AllerTOP/ (accessed on 1 December 2021)). The spatial structures of the peptides were predicted using the PEP-Fold3 tool (https://bioserv.rpbs.univ-paris-diderot.fr/services/PEP-FOLD3 (accessed on 1 December 2021)).

### 2.6. Antiproliferative Activity Prediction—In Vitro Study

#### 2.6.1. Cell Lines and Culture Conditions

Mouse fibroblast cell line NCTC clone 929 (L cell, L-929, derivative of Strain L; (ATCC^®^ CCL-1™) and colon carcinoma cell line CT-26 (N-nitroso-N-methylurethane-induced, undifferentiated colon carcinoma cells, cloned to generate the designated CT-26. WT cell line; ATCC^®^ CRL-2638™) were obtained from ATCC and cultured with the manufacturer’s instructions. Complete Eagle’s Minimal Essential medium (Pan-Biotech) and RPMI-1640 medium (Sigma-Aldrich) supplemented with 10% fetal bovine serum (Good HI, Sigma-Aldrich) and antibiotics (100 IU/mL penicillin, 10 mg/mL streptomycin, 25 µg/mL amphotericin B; Pan-Biotech) were used for culturing L-929 and CT-26 cell lines, respectively. Culturing was carried out in a Galaxy 170R incubator under controlled growth conditions, with constant humidity and 5% CO_2_. After multiplication and stabilization of the cells (approximately 7–14 days), when the culture reached at least 75% confluence, the cells were cultured with prepared variants in different concentrations.

#### 2.6.2. MTT Assay

This assay is based on the ability of mitochondrial succinate dehydrogenase to reduce the yellow tetrazolium salt (MTT) to purple formazan crystals. As this activity is carried out by living cells, the measured absorbance is directly proportional to the quantity of live cells [27,28]. After multiplication and stabilization of cells (approximately 7–14 days), the culture reached at least 75% of confluence; after reaching confluence, the tested preparations were added to the cell culture in the final concentration (2.5, 25, 50, 100, and 250 µg/mL) and incubated for 24, 48, and 72 h. After this time, the cells were subjected to the MTT assay (MTT Cell Proliferation/Viability Cell Assay: BIOKOM analysis) in triplicate. The control well contained only cells (L-929 or CT-26) without the test preparation. The absorbance was measured using a microplate reader, and absorbance was measured at 570 nm (MULTISCAN FC, Thermo Scientific).

#### 2.6.3. Statistical Analysis

Statistical analysis was carried out using Statistica 13.1 (StatSoft, Cracov, Poland). The differences between mean values were found to be significant if their P-value was <0.05 based on Tukey’s range t-test. The results are expressed as mean ± standard deviation graphically. In vitro analyses were performed in triplicate.

## 3. Results

### 3.1. Spectrometric Identification and In Silico Prediction of Potentially Anticancer Properties

**Figure 1:**
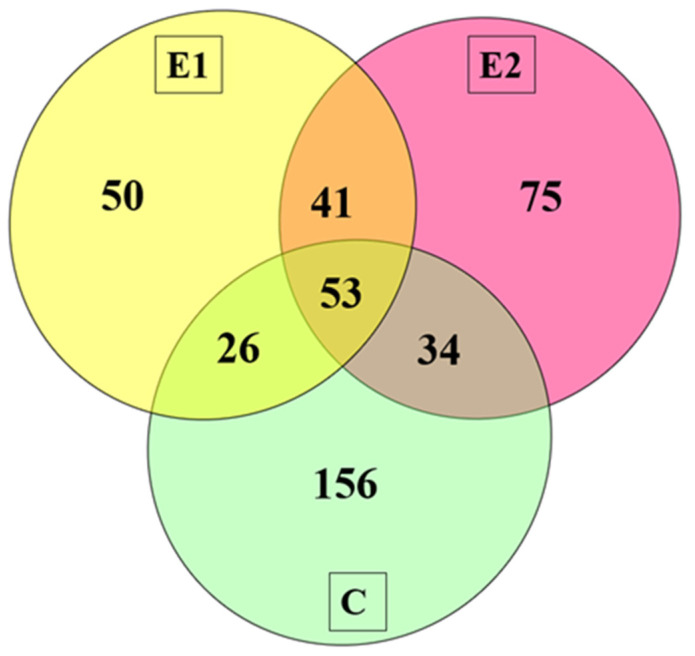
Venn diagram (E1 and E2—samples added with sodium nitrite at an amount of 50 mg/kg of stuffing and lyophilized willow herb extract at an amount of 150 and 1000 mg/kg, respectively; C—control sample added with sodium nitrite at an amount of 100 mg/kg of stuffing but not willow herbs.

The spectrometric analysis revealed the presence of 1626 peptide sequences in the canned meat extracts. The identified peptide sequences had a chain length of 7–37 amino acids with a lot of Lys, Ala, and Ile, and devoid of Cys and Trp. All the identified peptides were screened for fragments characterized by potentially anticancer activity. In E1 samples (containing extract at a concentration of 150 mg/kg), 21.46% of potentially anticancer fragments were determined (170 out of 792 sequences in total), while in E2 (containing extract at a concentration of 1000 mg/kg) 27.28% of potentially anticancer fragments were identified in the total pool of peptides (203 out of 744 sequences). In the control sample, 25.74% of potentially anticancer peptides were identified (269 out of 1045 sequences). Such a high percentage of potentially anticancer peptides found in canned pork after 180 days of storage indicates that the product is promising and may serve as a functional food for improving human health. Table 1 presents the sequences of potentially anticancer peptides identified (53 fragments) in all analyzed samples (common for E1, E2, and C). A qualitative comparison of unique and common peptides identified in the three study variants, as shown in Figure 1, indicated a greater similarity between the samples containing the willow lyophilizate (E1 and E2) than the control samples.

**Figure 2:**
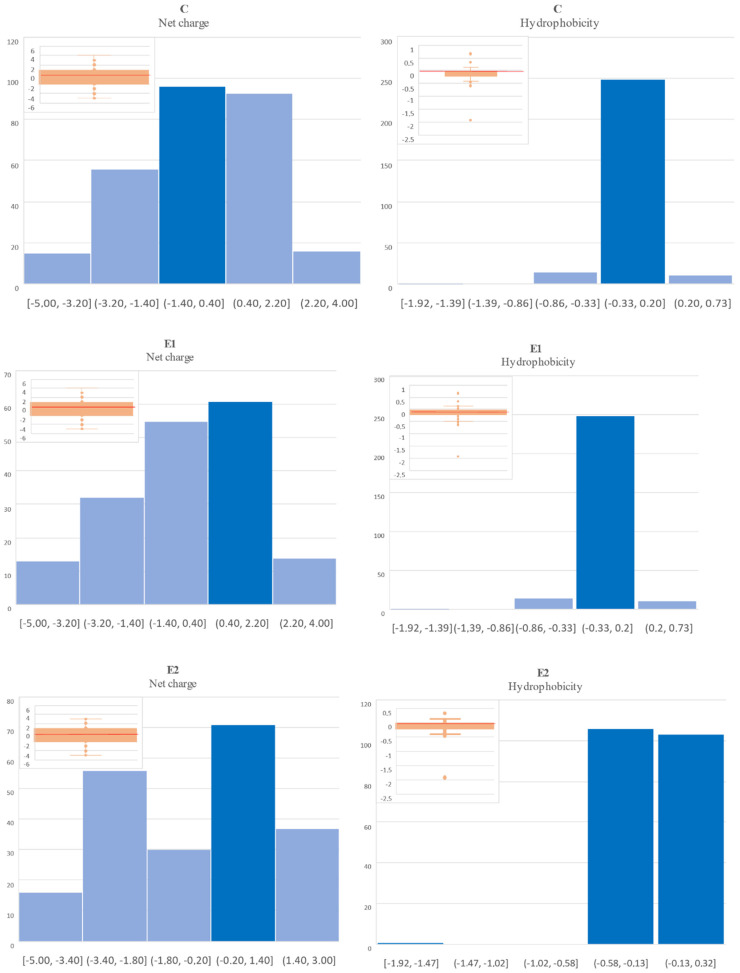
Summary of hydrophobic properties and net charge of all sequences of peptides with potentially anticancer activity derived from canned pork after 6 months of refrigerated storage (the y-axis shows the number of peptides with characteristic net charge or hydrophobicity values, whose specific ranges are represented by the x-axis in the above figures).

Figure 2 shows the values of the net charge distribution and the hydrophobicity of analyzed molecules. The net charge of peptides derived from the tested samples ranged from −5 to 4 (or 3 for E2), while most of the peptides had a net charge of around 0.0. For example, the net charge of LEDEVYAQKMKYKAISEELDNALNDITSL peptide from E1 was on average 0.017 with a minimum value of −5 and that of EALGDKAVFAGRKFRNPKAK was 4. The highest number of potentially anticancer peptides had a net charge of 0.4–2.2, which indicates that they were cationic peptides.

Taking into account hydrophobicity, most of the sequences detected in this study had a hydrophobicity value below 0, and most often less than −0.33 (Figure 2). This tendency was observed for all the analyzed variants. The hydrophobicity index values determined in this study did not allow the unequivocal classification of peptides, as well soluble or as poorly soluble, because the obtained values were both additive (but below +1) and negative (even below almost −2), and, in the majority of the cases, the value was close to 0 (zero) in each of the analyzed variants (Figure 2).

**Figure 3:**
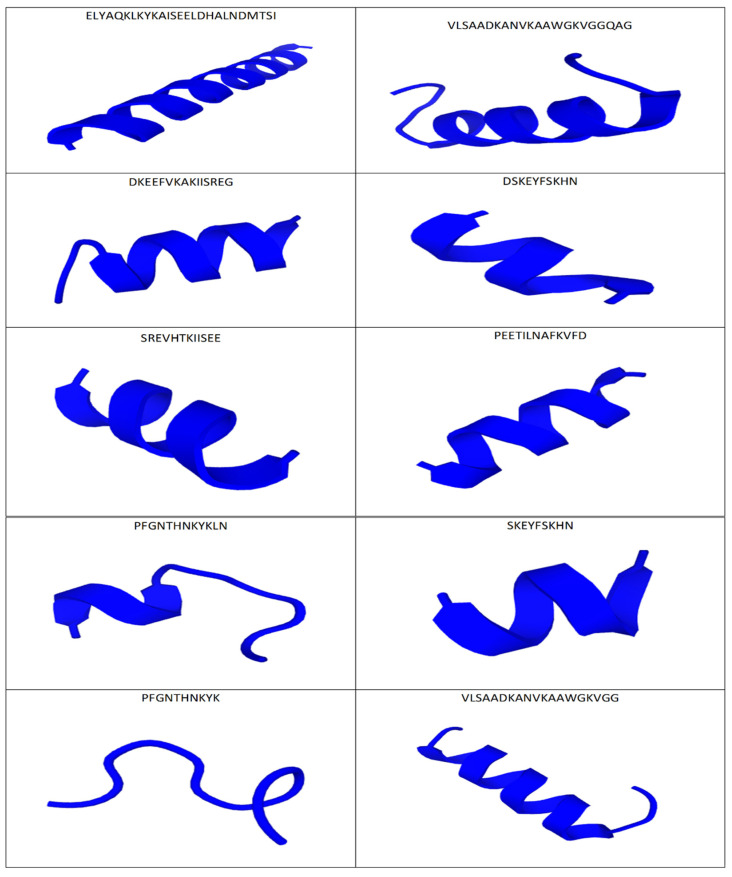
De novo 3D models of selected sequences of peptides with potentially anticancer activity derived from canned pork after 6 months of refrigerated storage irregular sequence—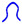, helix sequence—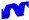.

To examine the effect of particles’ structure on their biological activity on cancer cells, putative antitumor peptides were visualized, and 10 of them with the highest index of “anticancer probability” are shown in Figure 3. All selected molecular structures of the peptides were predicted using the PEP-Fold3 tool. The conformation of the peptides indicated different structures, irrespective of the sequence size. The bioactive sequences were embedded only in the random coil (as was the case with PFGNTHNKYK), but most of the potentially anticancer peptides assumed the α-helical conformation (Figure 3).

In this study, an in silico analysis of the toxicity and allergenicity of the selected sequences was also performed. None of them were toxic. In contrast, half (51.4%) of the sequences included in the analysis were classified as “possibly allergen” and the other half (48.6%) as “possibly non-allergen”.

### 3.2. In Vitro Prediction of Antiproliferation Properties

**Figure 4:**
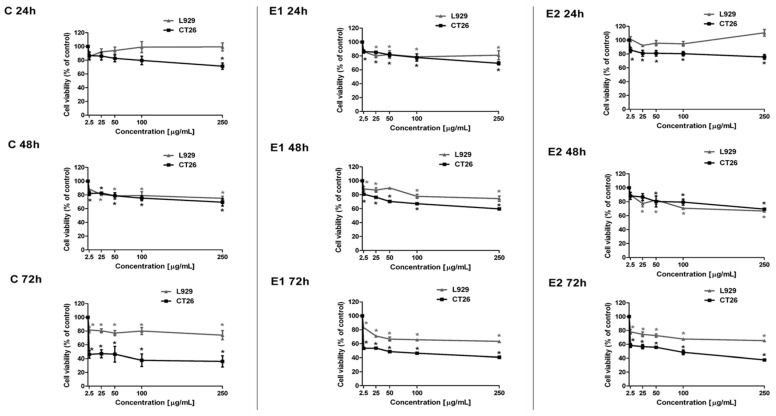
Results of antiproliferation activity in the in vitro analysis (E1 and E2—samples added with sodium nitrite at an amount of 50 mg/kg of stuffing and lyophilized willow herb extract at an amount of 150 and 1000 mg/kg, respectively; C—control sample added with sodium nitrite at an amount of 100 mg/kg of stuffing, but not willow herbs) (sign * means that the differences were statistically significant at *p* < 0.05).

For the in vitro analysis, the identified sequences were hydrolyzed with pepsin and pancreatin, to simulate the conditions of the human digestive system. The obtained sequences were assessed to ensure whether they were potentially antiproliferation candidates by determining their specificity of action (action against normal cells) and effectiveness against cancer cells. Therefore, after confirming by in silico analysis that canned meat can be a potential source of anticancer peptides, the activity of the experimental variants was examined in vitro by cell viability study using cell lines. The cultures of healthy fibroblast L-929 cells and colorectal carcinoma CT-26 cells were grown until confluence (above 75%) and then treated with the above-mentioned variants containing the tested extract at different concentrations (2.5, 25, 50, 100, and 250 µg/mL). The viability of cells was tested after incubating them with the preparations for 24, 48, and 72 h. As could be expected for the control sample, no particularly negative effect of the pure canned pork preparation on both cell types was noted, regardless of the concentration used. Only in neoplastic cells, a significant decrease in viability was observed after 72 h. In the case of the variant E1, after 24 h of incubation, a slight decrease in cell viability to about 80% was observed in both cell lines, for all concentrations of the preparation used, except for 250 µg/mL, which reduced the viability of CT-26 cells below 80%. The viability of normal L-929 cells remained relatively constant, i.e., about 80%, throughout the experiment after treatment with the E1 variant. On the other hand, cancer cells showed reduced viability regardless of the concentration of the E1 variant used. A decrease in cell viability to about 60% was observed after 48 h of incubation and to about 40% after 72 h, which is very promising (Figure 4). The data obtained for the E2 variant revealed its interesting effect on L-929 cells. After 24 h, an improvement in cell viability, up to around 110%, was noted for the E2 variant at the concentration of 250 µg/mL; at the same time, the appropriate concentration of E2 reduced the viability of CT-26 cells to about 80%. After 48 h of incubation, in both tested cell lines, the results were found to be analogous to the E1 variant. On the other hand, 72 h incubation with E2 reduced the viability of intestinal cancer cells to about 40%, and normal cells to only about 70%.

## 4. Discussion

### 4.1. Spectrometric Identification and In Silico Prediction of Potentially Anticancer Properties

The biological activity of food-derived peptides, including activity against cancer cells, is determined by their structural characteristics, such as amino acid composition, sequence, length, and total charge/hydrophobicity. Most food-derived anticancer peptides contain short amino acid sequences with residues ranging from 3 to 25 [29] or even longer [30], although peptides of lower molecular weight seem to have higher cytotoxicity [31]. In this study, the identified peptide sequences had a chain length of 7–37 amino acids, with the largest portion of fragments having 13–16 amino acids. Moreover, the presence of amino acids, such as Pro, Leu, Gly, and Ala, as well as one or more Lys, Arg, Ser, Glu, Thr, and Tyr residues, in the anticancer peptides can enhance their interactions with the tumor molecule, thus, exerting a cytotoxic effect on cancer cells [29,32]. Therefore, this study analyzed the amino acid composition of potentially anticancer peptides using the ProtParam bioinformatics tool. The analysis revealed the following relationships: Lys (11%) > Ala (10%) > Ile (9%) > Glu, Leu (8%) > Gly (7%) > Val, Asp, Ser (6%) > Thr (5%) > Pro, Asn (4%) > Phe, Met, Tyr (3%) > His, Arg, Gln (2%). The peptide sequences determined in the study contained neither Cys nor Trp. They consisted of hydrophobic residues, such as Ala, Ile, and Leu, but some polar residues, such as Lys (highest frequency) or Glu, were also found. The presence of Glu and Pro has been linked with anticancer properties of peptides [12,29], which strengthens our assumption about the anticancer activity of the peptides derived from canned meat. Additionally, the hydrophobic amino acids determine the overall hydrophobicity of peptides. Song et al. [31] reported that the Tyr-Ala-Leu-Pro-Ala-His peptide present in the hydrolysate of half-fin anchovy (Setipinnataty) showed antiproliferative activity on PC-3 cells (human prostate cancer), which was attributed to the presence of hydrophobic amino acids (about 50%), as well as the high net charge.

The structure, as well as net charge and hydrophobicity, which depends inter alia on the amino acid composition of the sequence, have an impact on the specificity of targeting and interaction of anticancer peptides with cancer cells. Thus, it is important to analyze these characteristics when assessing the anticancer potential of biological molecules. The net charge is a significant factor in the design of antimicrobial peptides, as it allows their interaction with tumor cells and determines their mechanisms of action against these cells [33]. As shown in Figure 2, the highest number of potentially anticancer peptides were cationic peptides (had a net charge of 0.4–2.2). Cationic peptides are positively charged and are capable of interacting with the phospholipids expressed on the surface of cancer cells, such as phosphatidylserine and phosphatidylglycerol, which carry a net negative charge and provide sites for electrostatic attraction between the peptides and cancer cells. Following electrostatic interaction, the pores of cell membranes are opened, allowing intracellular components to penetrate, which cause cell necrosis [34,35]. According to the literature, peptides that internalize and bind to the mitochondrial membrane destabilize it by activating an apoptotic pathway mediated by caspase release of cytochrome C and, consequently, apoptosis [36]. The net charge values determined in our study are of a wide range, which indicates the presence of cationic peptides (with a positive net charge), although there were also sequences with a negative net charge. In this study, the highest net charge value of +4 was found for peptides from E1 and C samples. This indicates that they may be candidates with anticancer potential, as Ntwasa et al. [37] showed that cationic peptides with charges between +2 and +9 usually interact better with the anionic heads of phospholipids.

Taking into account the hydrophobicity index, the obtained values were quite diverse. This result can be due to the presence of specific amino acids that make up the individual peptide sequences, which included, as described above, both hydrophobic and hydrophilic amino acids.

Moreover, the amino acid composition directly influences the structure of peptide fragments. The literature data suggest that the structural configuration of peptides influences their functions, especially those related to the destruction of pathogenic and/or neoplastic cells [35]. The typical configurations of anticancer peptides were extended and spatial structures (α-helix or β-sheet) [38]. These structures, which are amphipathic in nature, mainly consist of a cationic and hydrophobic surface, which facilitates the interaction of the peptide with the target cell [38]. They were also dominant among the peptides derived from canned pork after 180 days of storage (Figure 3), contributing to their potential anticancer effect.

Despite non-toxicity, an unresolved issue is the allergenicity of the proposed peptides from meat product with dandelion extract. In silico analyses is based on the AllerTOPv. 2.0 tool. This bioinformatics tool is a robust and complementary tool developed based on the k-nearest neighbor method for the classification of allergens and non-allergens. It has an accuracy of 88.7% and is the most effective for in silico allergen prediction [39]. However, it should be noted that in silico methods can only determine whether a new protein is an existing allergen or whether it may cross-react with an existing allergen and not whether the new protein will “become” an allergen [40]. Furthermore, as pointed out by Hayes et al. [41], the predictive value of sequence similarity search should be carefully considered when determining allergenicity potential, as in silico results do not perfectly correlate with the occurrence of food allergy. Moreover, the relatively high degree of amino acid sequence-level identity often observed between cross-reactive IgE proteins cannot guarantee that the protein is a cross-reactive allergen [42]. Thus, a high level of potential allergenicity or no allergenicity determined in this study may not be an indication of allergy, but only draw attention to a potentially health problem. Further biological, biochemical, or in vivo tests should be performed to confirm the allergenicity prediction of peptides derived from canned pork containing freeze-dried willow extract. In particular, the hydrolysates that were proven to be sensitizing by in silico analysis may turn out to be unstable not only in the intestine but also in the vascular system or the liver. Therefore, bioactive peptides must be resistant to enzymes in the human digestive system and remain stable in the further regions of their distribution in the human body up to the site of action. In fact, digestion in the gastrointestinal tract promotes the release of biopeptides from large sequences of food proteins, resulting in the release of biologically active fragments, which, when digested, and absorbed in the small intestine, retain their biological properties. On the other hand, intense hydrolysis can cause the destruction of peptide sequences, reducing their biological activity.

### 4.2. In Vitro Prediction of Antiproliferation Properties

The approach of assessing the viability of peptides against cell lines, as used in this study, has already been successfully applied by other researchers. Various tumor cell lines, such as MCF-7, MDA-MB- 231, and BT549 (human breast cancer); Ca9-22 and CAL 27 (human oral cancer); Hep G2 (human liver cancer); HT-29, RKO, KM12L4, DLD-1, and HCT15 (human colon cancer); Caco-2, TC7, and HCT-116 (human colon cancer); U87 (human glioma); PC3, LNCaP, and DU-145 (human prostate cancer); THP-1 (human monocytic leukemia); Jurkat T cells (human T cell leukemia); AGS (human gastric cancer); A549 and H-1299 (human lung cancer); HeLa (human cervical cancer); 109 (human esophageal cancer); hFOB1.19 (human fetal osteoblastic carcinoma); HL-60 (human acute myeloid leukemia); Kelly, SK-N-DZ, and IMR-32 (human neuroblastoma); L1210 (murine lymphocytic leukemia); P388D1 (mouse monocyte cell line); MC3T3E1 (mouse osteoblastoma), UMR106 (rat osteosarcoma); and vero (monkey kidney cancer cells), have been widely used as model cell culture systems to investigate the antitumor effects of protein hydrolysates prepared from different sources of dietary protein [32]. For example, Ito et al. [43] reported that porcine skin gelatin showed in vitro antitumor effects on murine hepatoma cells (MH134), inducing programmed cell death (apoptosis). Similarly, Castro et al. [44] confirmed the effect of bovine collagen hydrolysates (BCH) on the proliferation of B16F10 melanoma cells. In turn, Daliri et al. [45] analyzed the effect of bioactive peptides isolated from soybean, oyster, and sepia ink hydrolysates, rapeseed protein fermentates, and tuna cooking juice on various cancer cell lines. Other authors [46,47] observed that bioactive peptides derived from bovine sarcoplasmic proteins had a positive effect on breast and gastric cancer cells. Apart from the potential anticancer properties of peptides, the secondary products of willow herb might also be beneficial to human health. Oenothein B is the main substance found in Epilobium species, which is considered to have anticancer properties [48]. Therefore, their application in food may have a positive effect on health [45]. Indeed, in presented study, in vitro analyses on L-929 (mouse fibroblast cell line) and CT-26 (undifferentiated colon carcinoma cell line) confirmed the antitumor activity of peptides obtained from the meat product. The results of own research on cell lines indicate the anticancer potential of peptide from canned meat with willow extract. Particularly noteworthy is the fact that after 72 h of incubation with the peptides obtained from the variant containing 1000 mg/kg of freeze-dried willow herb extract, the viability of intestinal cancer cells was decreased to about 40%, while by comparison the viability of normal cells was decreased to only about 70%. The promising results observed for canned pork added with freeze-dried willow extract suggest the usefulness of the herb in the production of meat products with nutritional, as well as health benefits. 

## 5. Conclusions

This study analyzed the potentially anticancer activity of meat products prepared with freeze-dried willow herb extract. Among the identified peptide sequences, some were cationic and hydrophobic with α-helical or β-sheet conformation, which indicates their anticancer potential. Furthermore, the sequences of peptides present in the proteolytic enzyme hydrolysates showed, on the one hand, the resistance of these peptides to simulated conditions of the human digestive system, and, on the other hand, their preserved biological activity was confirmed by their action on the tested cell lines. Thus, the results of the study shed new light on the value of canned meat as a functional food, due to its complementing nutritional and physiological properties. However, there is a need for further research on anticancer peptides, especially in terms of their selectivity or their half-life in the body and the exclusion of potential allergenicity.

## Figures and Tables

**Table 1:** List of potentially anticancer peptide sequences found in all the analyzed variants.

No.	Peptide Sequences	Protein Source	Anticancer Probability *	No.	Peptide Sequences	Protein Source	Anticancer Probability *
1	PFGNTHN	Creatine kinase M-type	0.9596	28	VLSAADKANVKAAWGKVGGQAG	Hemoglobin subunit alpha	0.9946
2	LVKAGFAGD	Actin, alpha skeletal muscle	0.9085	29	PPFEVRGANQWIKFKSIS	Pyruvate dehydrogenase E1 component subunit alpha	0.9560
3	SKEYFSKHN	Peroxiredoxin-2	0.9934	30	SEIQNIKSELKYVPRAEQ	Small muscular protein	0.5636
4	VSTVLTSKYR	Hemoglobin subunit alpha	0.5404	31	VQAAFQKVVAGVANALAHKYH	Hemoglobin subunit beta	0.6455
5	VLSAADKANVKA	Hemoglobin subunit alpha	0.9699	32	GELAKHAVSEGTKAVTKYTSSK	Histone H2B type 3-B	0.8512
6	PFGNTHNKYK	Creatine kinase M-type	0.9959	33	VTGNLDYKNLVHIITHGEEKD	Myosin regulatory light chain 2	0.8801
7	VITHGDAKDQE	Myosin regulatory light chain	0.9596	34	PIIQDRHGGYKPTDKHKTDLN	Creatine kinase M-type	0.9860
8	DSKEYFSKHN	Peroxiredoxin-2	0.9944	35	EVYAQKMKYKAISEELDNALNDITSL	Tropomyosin beta chain	0.9880
9	PEDVITGAFKVL	Myosin regulatory light chain 2	0.7486	36	ELYAQKLKYKAISEELDHALNDMTSI	Tropomyosin alpha-3 chain	0.9991
10	KVEELKKKYGI	Acyl-CoA-binding protein	0.9708	37	LEDEVYAQKMKYKAISEELDNALNDITSL	Tropomyosin beta chain	0.9110
11	LVHIITHGEEKD	Myosin regulatory light chain 2	0.8112	38	LEDELYAQKLKYKAISEELDHALNDMTSI	Tropomyosin alpha-3 chain	0.9577
12	PEDVITGAFKVLD	Myosin regulatory light chain 2	0.8566	39	DLEDEVYAQKMKYKAISEELDNALNDITSL	Tropomyosin beta chain	0.9228
13	SREVHTKIISEE	Myosin-1	0.9965	40	DLEDELYAQKLKYKAISEELDHALNDMTSI	Tropomyosin alpha-3 chain	0.9625
14	PFGNTHNKYKLN	Creatine kinase M-type	0.9965	41	ILAADESTGSIAKRLQSIGTEN	Fructose-bisphosphate aldolase	0.9424
15	NLVHIITHGEEKD	Myosin regulatory light chain 2	0.7555	42	PVVQAAYQKVVAGVANALAHKYH	Hemoglobin subunit beta	0.5353
16	PEETILNAFKVFD	Myosin regulatory light chain 2	0.9940	43	PEDVITGAFKVLDPEG	Myosin regulatory light chain 2	0.7505
17	PFGNTHNKYKLNF	Creatine kinase M-type	0.8819	44	KYKAISEELDNALNDITSL	Tropomyosin beta chain	0.7309
18	SADTLWGIQKDLKDL	L-lactate dehydrogenase B chain	0.9896	45	DKEEFVKAKIISREG	Myosin-7	0.9997
19	AISEELDHALNDMTSI	Tropomyosin alpha-3 chain	0.8959	46	DKEEFVKAKILSRE	Myosin-7	0.9808
20	YKNLVHIITHGEEKD	Myosin regulatory light chain 2	0.6486	47	VAGDEESYVVFKDLFD	Creatine kinase M-type	0.5149
21	PEDVITGAFKVLDPEGK	Myosin regulatory light chain 2	0.8019	48	PEDVITGAFKVLDPEGKGT	Myosin regulatory light chain 2	0.8671
22	VLSAADKANVKAAWGKVGG	Hemoglobin subunit alpha	0.9934	49	LAKHAVSEGTKAVTKYTSSK	Histone H2B type 1-N	0.9295
23	FQKVVAGVANALAHKYH	Hemoglobin subunit beta	0.7132	50	DEVYAQKMKYKAISEELDNALNDITSL	Tropomyosin beta chain	0.9898
24	PEDVITGAFKVLDPEGKG	Myosin regulatory light chain 2	0.8395	51	AKLKEIVTNFLAGFEA	ATP synthase subunit alpha	0.5994
25	FGPTGIGFGGLTHQVEKKE	Cysteine- and glycine-rich protein 3	0.5000	52	AMKAYINKVEELKKKYGI	Acyl-CoA-binding protein	0.7171
26	KAKDIEHAKKVSQQVSKV	Nebulin	0.5793	53	VLSAADKANVKAAWGKVGGQAGAH	Hemoglobin subunit alpha	0.9858
27	LDYKNLVHIITHGEEKD	Myosin regulatory light chain 2	0.8578				

* Analysis carried out using the iACP tool, in which the probability of biological activity was determined on a scale of 0 (0% activity) to 1 (100% activity).
